# Supplementary material for: Canopy Size and Light Use Efficiency Explain Growth Differences between Lettuce and Mizuna in Vertical Farms
Source: Plants (Basel). 2021 Apr 6;10(4):704. doi: 10.3390/plants10040704 (PMC8067451; doi:10.3390/plants10040704)
Supplement: Supplementary file 1 [file plants-10-00704-s001.pdf]

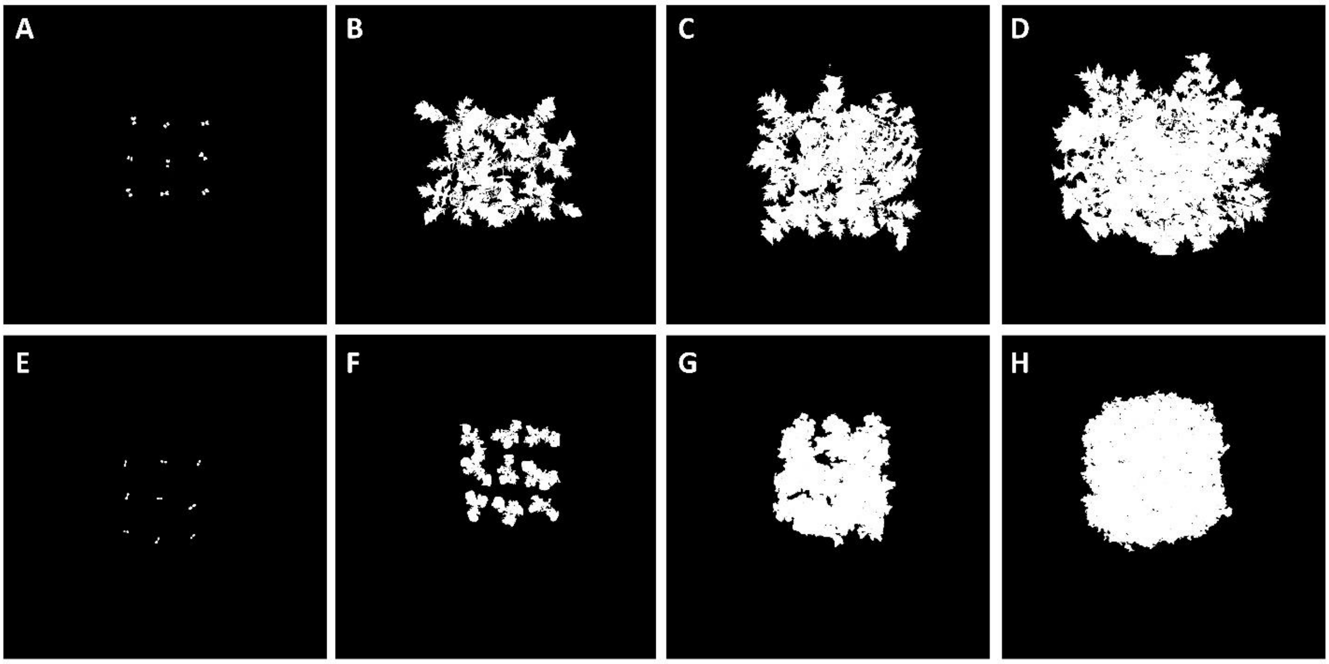

Figure S1. The projected canopy images captured during the growing period of mizuna (*Brassica rapa* var. *japonica*) (A–D) and lettuce (*Lactuca sativa* 'Green Salad Bowl') (E - H) grown at 425 μmol m^{-2} s^{-1}The first three images of mizuna (A–C) and lettuce (E–G) were captured at 5 (A, E), 15 (B, F), and 19 days (C,G) after seeding. The last image of mizuna (D) and lettuce (H) were captured during the harvest (27 and 28 days, respectively).

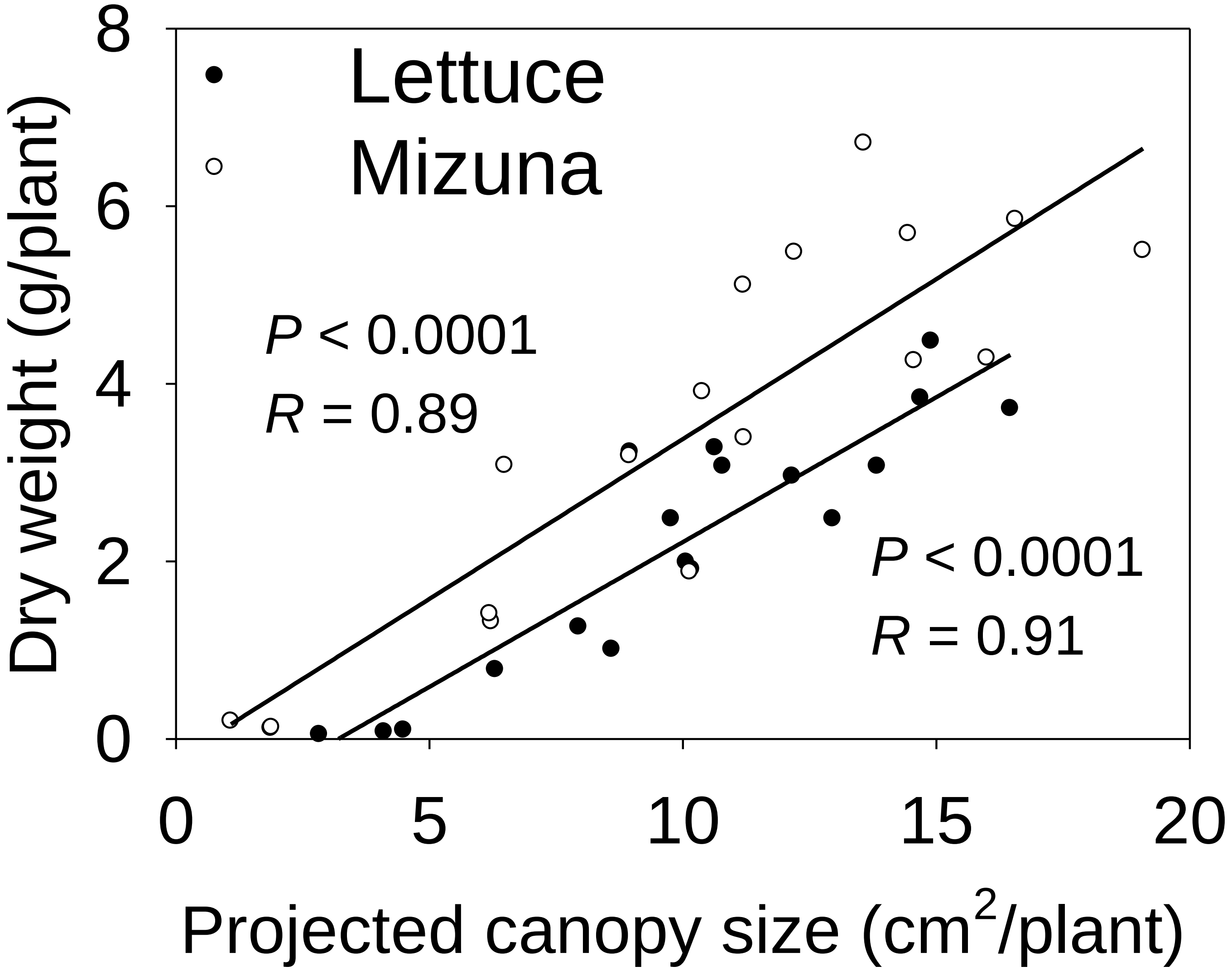

Figure S2. Correlation between early projected canopy size (lettuce at 10 days and mizuna at 8 days, after seeding) and final shoot dry weight of lettuce (*Lactuca sativa* 'Green Salad Bowl') and mizuna (*Brassica rapa* var. *japonica*).

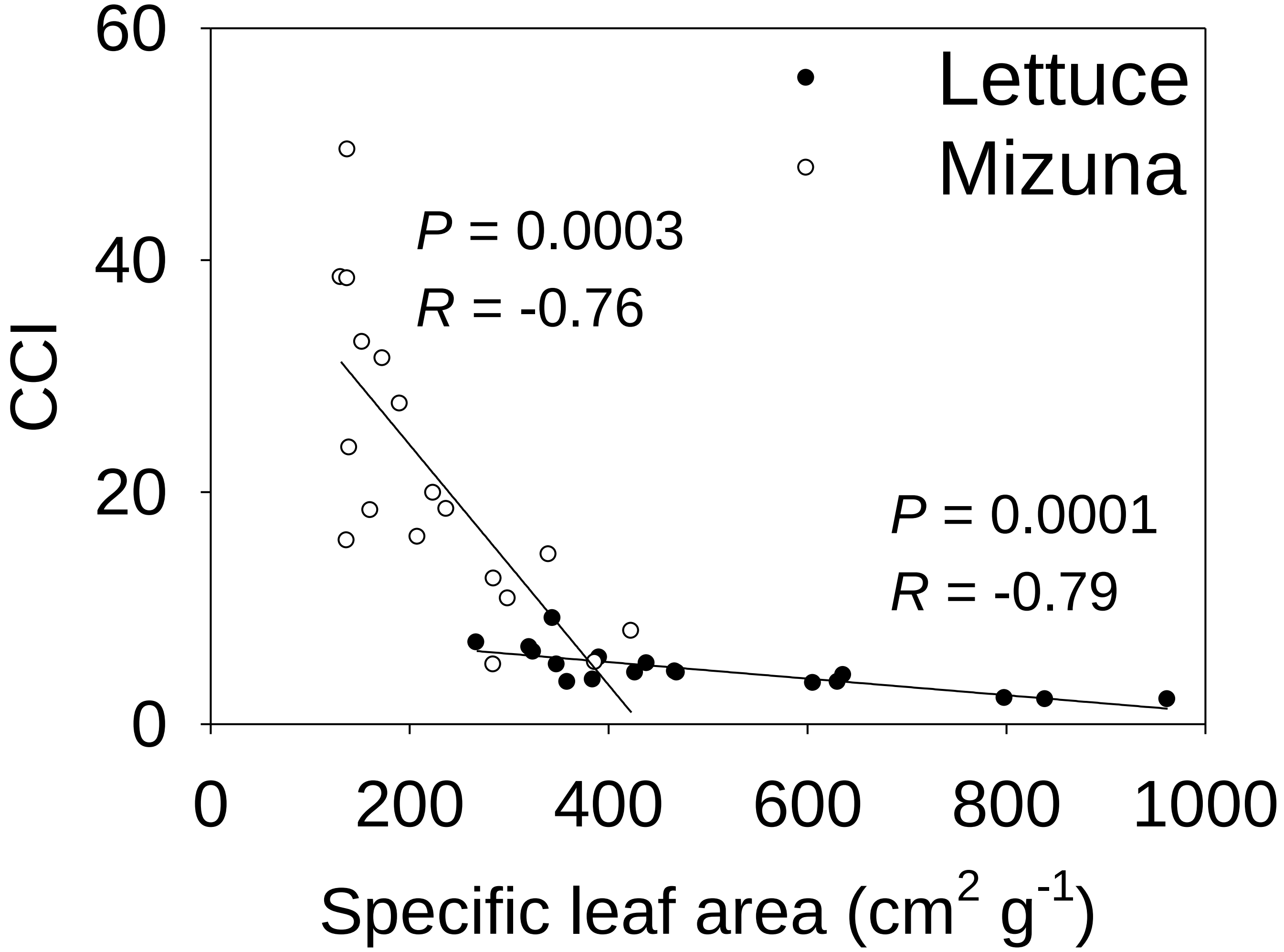

Figure S3. Correlation between specific leaf area (leaf area / shoot dry weight) and chlorophyll content index (CCI) of lettuce (*Lactuca sativa* 'Green Salad Bowl') and mizuna (*Brassica rapa* var. *japonica*).

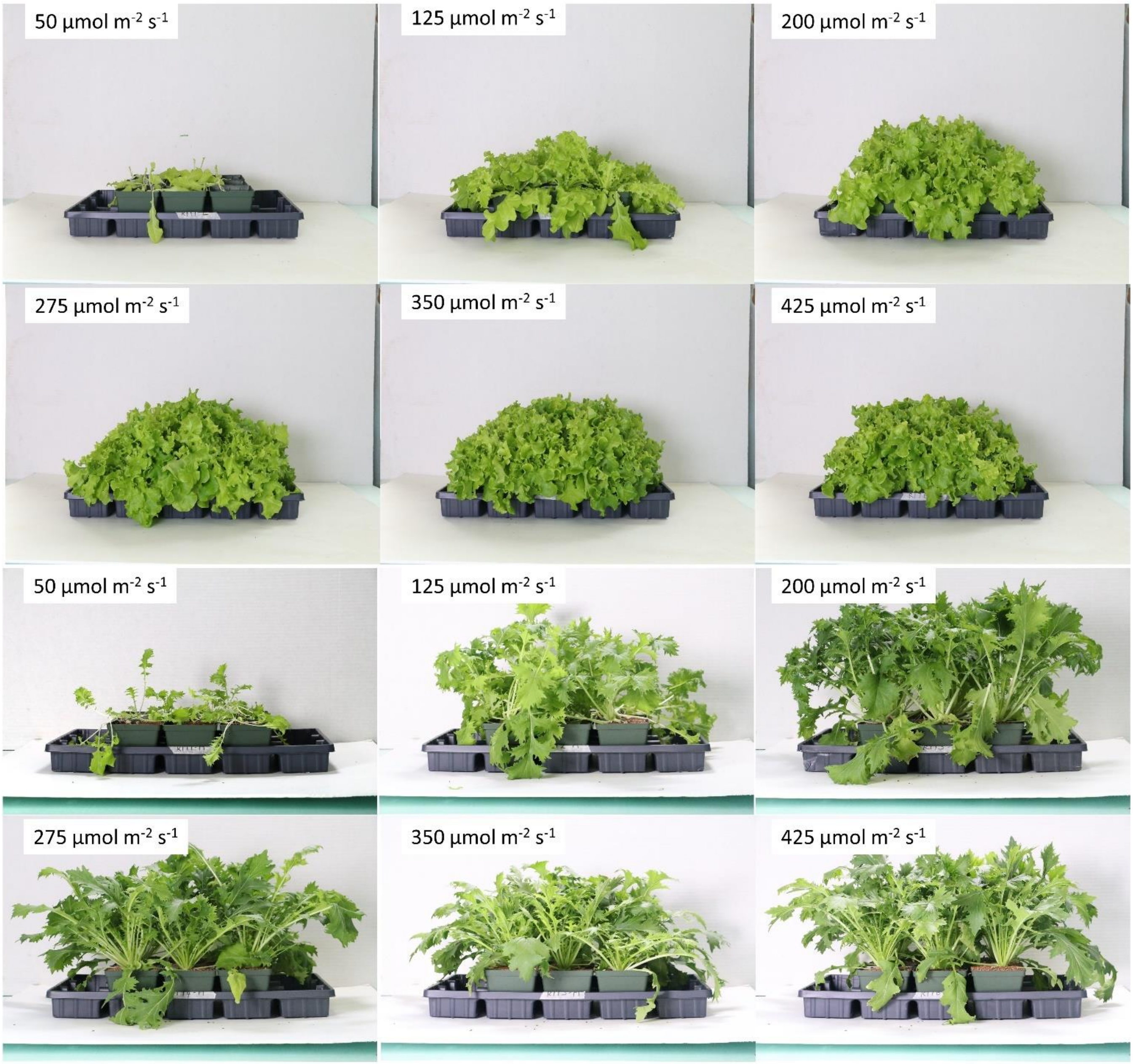

Figure S4. The appearance of lettuce (*Lactuca sativa* 'Green Salad Bowl', top) and mizuna (*Brassica rapa* var. *japonica*, bottom) at the end of the growing cycle for plants grown at six different photosynthetic photon flux densities (upper left corner of each picture).
